# Collapse of an iconic conifer: long-term changes in the demography of *Widdringtonia cedarbergensis* using repeat photography

**DOI:** 10.1186/s12898-016-0108-6

**Published:** 2016-11-30

**Authors:** J. D. M. White, S. L. Jack, M. T. Hoffman, J. Puttick, D. Bonora, V. Visser, E. C. February

**Affiliations:** 1 Plant Conservation Unit, University of Cape Town, Cape Town, South Africa; 2 Department of Biological Sciences, University of Cape Town, Cape Town, South Africa; 3 Statistics in Ecology, Environment and Conservation, Department of Statistical Sciences, University of Cape Town, Cape Town, South Africa; 4 African Climate and Development Initiative, University of Cape Town, Cape Town, South Africa

**Keywords:** Temperature, Conifer, Repeat photography, Population change, Climate change, Fire, Cederberg

## Abstract

**Background:**

Conifer populations appear disproportionately threatened by global change. Most examples are, however, drawn from the northern hemisphere and long-term rates of population decline are not well documented as historical data are often lacking. We use a large and long-term (1931–2013) repeat photography dataset together with environmental data and fire records to account for the decline of the critically endangered *Widdringtonia cedarbergensis.* Eighty-seven historical and repeat photo-pairs were analysed to establish 20th century changes in *W. cedarbergensis* demography. A generalized linear mixed-effects model was fitted to determine the relative importance of environmental factors and fire-return interval on mortality for the species.

**Results:**

From an initial total of 1313 live trees in historical photographs, 74% had died and only 44 (3.4%) had recruited in the repeat photographs, leaving 387 live individuals. Juveniles (mature adults) had decreased (increased) from 27% (73%) to 8% (92%) over the intervening period. Our model demonstrates that mortality is related to greater fire frequency, higher temperatures, lower elevations, less rocky habitats and aspect (i.e. east-facing slopes had the least mortality).

**Conclusions:**

Our results show that *W. cedarbergensis* populations have declined significantly over the recorded period, with a pronounced decline in the last 30 years. Individuals that established in open habitats at lower, hotter elevations and experienced a greater fire frequency appear to be more vulnerable to mortality than individuals growing within protected, rocky environments at higher, cooler locations with less frequent fires. Climate models predict increasing temperatures for our study area (and likely increases in wildfires). If these predictions are realised, further declines in the species can be expected. Urgent management interventions, including seedling out-planting in fire-protected high elevation sites, reducing fire frequency in higher elevation populations, and assisted migration, should be considered.

**Electronic supplementary material:**

The online version of this article (doi:10.1186/s12898-016-0108-6) contains supplementary material, which is available to authorized users.

## Background

In a recent global review of forest tree species, climate-induced physiological stress driven by drought and high temperatures was found to be a major cause of mortality in the last 40 years [1]. Affected trees were also more vulnerable to climate-mediated processes such as insect or pathogen attack and wildfires. Conifers represented more than 40% of the 88 reviewed cases of mortality [1] and have recently been found to be particularly vulnerable to a warming climate [2].

The particularly rapid decline of the charismatic southern African endemic, *Widdringtonia cedarbergensis* Marsh [3] over the last century is representative of this global decline in conifers and has led the species to be listed as critically endangered under the current IUCN Red List of Plants [4]. While the diminishing number of *W. cedarbergensis* individuals have been anecdotally noted since the early 1800 s [5] and the focus of more systematic observation over the last 50 years (see [3]), there is as yet no quantitative evidence documenting the rate of decline and no plausible hypothesis for a cause.

The prevailing causal theories for the decline in *W. cedarbergensis* are (a) 18th and 19th century over-exploitation of the tree as a timber source which reduced and fragmented populations, thereby increasing ‘edge-effects’ and the likelihood of succumbing to fire [6–8], (b) late Quaternary climate change with warmer temperatures and less winter precipitation leading to a shift in the composition of co-occurring species with consequent changes in the fire regime [9, 10], (c) recent anthropogenic climate change, leading to temperature increases and aridity in the north-western areas of the fynbos biome, thereby increasing *W. cedarbergensis* mortality [11], and (d) the negative influence of insects and pathogens on reproductive output and survival [12].

The vegetation for the Cederberg Wilderness Area (CWA) is termed fynbos, a fire-prone vegetation type that includes many fire-adapted species with a fire return interval of approximately 11–15 years [13]. Fire is an important disturbance mechanism in fynbos and is thought to have had a strong influence in shaping the ecology and evolution of fynbos species [14]. *W. cedarbergensis,* however, is conspicuous in this environment in that it shows little or no adaptation to cope with fire. Mature trees are often killed by fire and display no re-sprouting ability, there is no canopy-stored seedbank, sapling growth rates are relatively slow, and the trees take much longer to reach reproductive maturity (ca. 40 years) than any co-occurring shrubs or trees [15]. Given such sensitivity to fire, an unfavourable fire regime can be very detrimental to this species. More recently there has been an increase in fire frequency with as many as six fires occurring in some parts of the Cederberg between 1977 and 2003 [16]. This suggests that the current fire return interval could be too short to allow for adequate establishment and reproductive output to maintain viable populations of the species [9, 13].

*Widdringtonia cedarbergensis* individuals are usually associated with rocky cliffs, outcrops and east facing slopes at high elevations and are rarely found at lower elevations, on flatter ground and deeper soils [3, 17]. This association with higher-lying rocky sites has not only been attributed to temperature amelioration [7] and protection from fire [3], but has also been suggested to give trees reliable access to available water trapped between bedding planes [11]. This water supply is reliant on regular rainfall, and therefore any change in the amount or seasonality of rainfall that may lead to a decline in plant available water will adversely affect the trees. Given that the CWA is projected to be one of the first areas in the Western Cape Province in South Africa to be affected by warming and drying due to anthropogenic climate change [18], the likely sensitivity of *W. cedarbergensis* to elevated temperatures and reduced moisture levels is concerning.

This study draws on a large collection of historical photographs of *W. cedarbergensis* (ca. 1931–1951 and 1960–1987) which were relocated and retaken (2007–2013), and represent the longest visual record of change in *W. cedarbergensis* populations to date. Here we (a) describe the pattern of recruitment and mortality of the species for the last 80 years, (b) evaluate the relationship between the degree of observed mortality and several likely environmental contributors to this mortality, and (c) speculate on the extent to which short-versus long-term changes in rainfall, temperature and fire regimes have affected the current distribution and demography. We use our findings to make recommendations regarding the future conservation of the species.

## Methods

### Study area and photograph sites

*Widdringtonia cedarbergensis*, commonly known as the Clanwilliam cedar, is endemic to the Cederberg Mountains of the Western Cape Province in South Africa. It has a patchy distribution between 900 and 1500 metres above sea level over approximately 250 km^2^, and typically grows in quartz-derived sandstone soils [19]. The Cederberg experiences a Mediterranean-type climate with the majority of frontally-derived rainfall received during the cooler winter months (April–September), while summers are hot and dry. Cederberg vegetation is comprised mainly of Cederberg Sandstone Fynbos, which is a sclerophyllous and fire-prone vegetation type [20, 21].

**Fig. 1:**
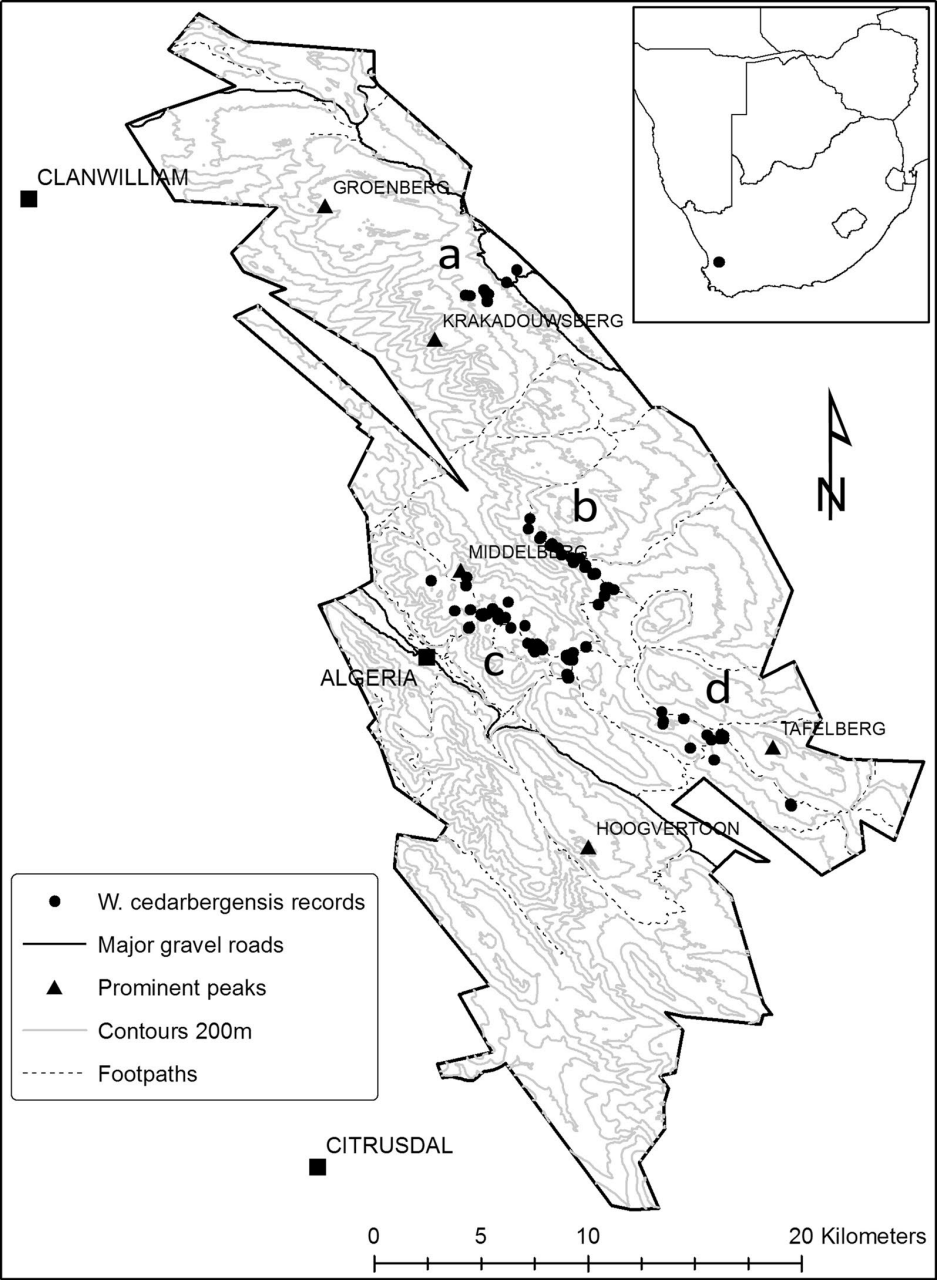
Map of localities of sampled *Widdringtonia cedarbergensis* individuals. The locations of *W. cedarbergensis* individuals in four different regions in the Cederberg Wilderness Area, Western Cape, South Africa: *a* Heuningvlei, *b* Skerpioenspoort, *c* Middelberg and *d* Welbedacht. Features of interest include the weather stations at Algeria Forest Station and Clanwilliam

At the elevations at which it grows, *W. cedarbergensis* is often the only tree species in the landscape. It is also uniquely different in growth form from the only other tree, *Protea nitida* and therefore easily distinguished. A historical collection of 87 photographs unambiguously showing *W. cedarbergensis* trees, taken between 1931 and 1987 by several photographers, were used in the analysis. Historical photographs were located in the Skerpioenspoort, Middelberg, Welbedacht and Heuningvlei regions and were repeated during three separate visits to the Cederberg in 2007, 2012 and 2013 (Fig. 1; see Additional file 1: Table S1 for further information on repeat photographs). In order to retake the historical photograph, sites were first located using a combination of topographical maps, notes accompanying photographs and landscape features. The exact position of the original camera was then determined in the field and, with the aid of a tripod for stability, the repeat photograph was taken using a high specification digital camera (Canon 5D MkII, Canon Inc., USA). Detailed site information, such as GPS co-ordinates, elevation, aspect, degree of rockiness, general ecological description and description of major changes in *W. cedarbergensis* and other vegetation, were recorded for each site.

### Population dynamics over time

**Fig. 2:**
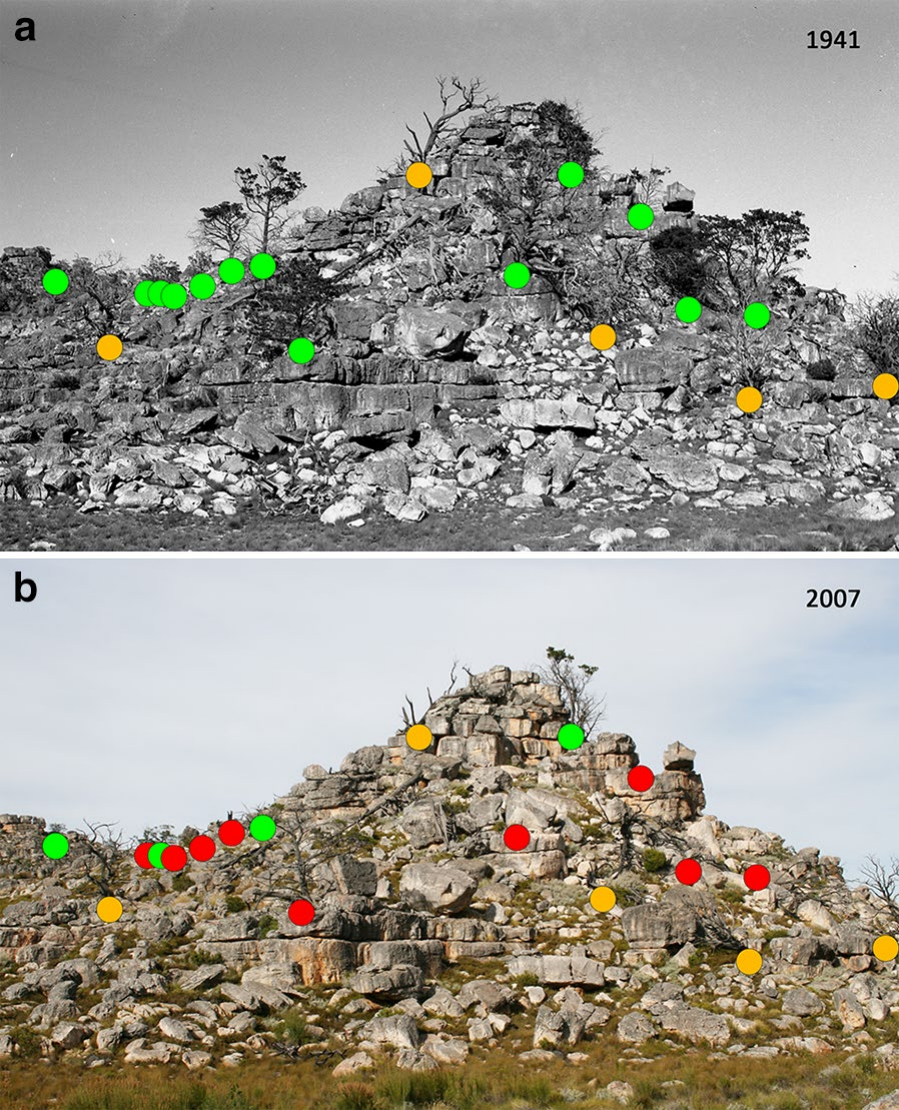
Example photo-pair. **a** Historical photograph taken by Ken Howes-Howell in 1941 and **b** repeat photograph taken by Timm Hoffman in 2007 at Vogelgesang, Skerpioenspoort. Thirteen living *Widdringtonia cedarbergensis* (*green dots*) and five skeletons (*orange dots*) were recorded in the historical photograph. Only four individuals had survived until 2007, with nine having died (*red dots*) and no new recruits

To match the photo-pairs, the repeat photograph was resized so that a distance between two fixed features common to both images was identical. The repeat photograph was then overlain on the historical photograph and rotated and shifted so that landscape features aligned exactly. The matched historical and repeat photographs were then saved independently (Adobe Systems Inc., 2010) and individual trees labelled according to whether these were living, dead or had recruited since the historical photograph was taken (Fig. 2). Mortality and recruitment probabilities were then calculated relative to the digitised original historic image. Age class (juvenile, mature adult and senescent/dead) and degree of rockiness were subjectively scored in both historical and repeat photographs. A juvenile tree was estimated to be generally shorter than 2 m tall with a thin, erect stem and no branching of the primary trunk. A mature adult was usually taller than a juvenile, with a thick primary trunk and a considerable extent of secondary branching, while senescent/dead individuals had only one or two branch tips with foliage or no foliage at all. Depending on the degree of rockiness, photograph sites were classified as either open (<25% rockiness), protected (25% < rockiness < 75%) or well protected (>75% rockiness and located within a rocky outcrop). Age class structure was then analysed within three broad time periods, namely 1931–1951 and 1960–1987 (historical photographs), and 2007–2013 (repeat photographs). These time periods, hereafter referred to as ‘early’, ‘middle’ and ‘recent’ respectively, were then compared using Chi squared tests, while the number of trees per photograph was compared for these periods using the nparcomp package in R (version 3.1.0) [22, 23].

### Environmental trends

The available fire history for the CWA was accessed via the SANBI Biodiversity GIS portal (http://bgis.sanbi.org), and fire frequency computed for each of the 87 photo-sites and identified regions [24]. Using GPS co-ordinates for each tree, mean annual temperature (°C) and annual precipitation (mm) values for the period 1950–2000 were extracted from the interpolated Worldclim climate surface [25]. The spatial resolution (30 arc sec) of WorldClim data did not allow for quantifications of temperature and precipitation for each tree. This is similarly the case for fire frequency data, where the temporal resolution did not exactly match the date of all historical photographs, while the myriad micro-habitats that may shelter plants from fires could not be mapped within fire ‘polygons’. However, the model variables represent the best available spatial and temporal estimates for climatic conditions presently available. Google Earth was used to determine latitude, longitude and aspect value for each tree [26]. Primary cardinal directions and intercardinal ranges were grouped for ease of analysis (for example: 315° < N ≤ 45°). Further information on the source, resolution and unit of measurement of modelled variables can be found in Additional file 2: Table S2. For climate trend analysis, historical climate data was obtained from the South African Weather Service for Clanwilliam (32°10′52.52″S, 18°53′37.64″E) and the Agricultural Research Council for the Cape Nature Offices at Algeria (32°22′28.65″S, 19° 3′38.34″E) (Fig. 1). Clanwilliam and Algeria were the closest stations with long-term precipitation records (1870–2010 and 1908–2008, respectively). No temperature records exist for Algeria while Clanwilliam has a temperature record from 1963 to 2010. We used the ‘segmented’ package in R to determine whether regression models of the precipitation and temperature records had unknown break-points [23, 27].

### Regional *W. cedarbergensis* population dynamics

All statistical analyses were performed in the R statistical programme [23]. For each region, defined as discrete, geographically separate populations, probability of mortality (Model 1) or recruitment (Model 2) was determined by fitting a generalized linear mixed-effects model (GLMM) in the ‘MASS’ package [28]. Habitat, aspect, mean annual temperature (°C) and fire frequency were fitted as fixed effects, while region and photograph site were fitted as random effects. This analysis included both plantations established at the end of the 19th century, as well as natural stands of trees. These models were simply used to describe regional differences in mortality and recruitment between *W. cedarbergensis* populations and not to infer abiotic correlates of population dynamics.

### Correlates of *W. cedarbergensis* tree mortality

Only natural stands were used in the analysis to determine environmental and climatic correlates of *W. cedarbergensis* mortality (Model 3). This model exhibited significant positive spatial autocorrelation for spatial lags up to about 5 km (measured using spatial correlograms from the ‘ncf’ package [29]; see Additional file 3: Figure S1). To account for spatial autocorrelation we included a spatial autocovariate using an exponential correlation structure with latitude and longitude coordinates. The GLMM used to determine correlates of mortality included mortality as the binary response variable, habitat, aspect, mean annual temperature (°C) and fire frequency as fixed effects and region and photograph site as random effects with a binomial family link. We used Wald tests to determine overall significance of the fixed effects [30].

## Results

### Population dynamics

A total of 1313 living trees were recorded when plantations and natural populations were combined across all historical photographs. Of these, 967 individuals (74%) had died and there were 44 new recruits (Heuningvlei: n = 2, Welbedacht: n = 9, Skerpioenspoort: n = 3 and Middelberg: n = 30), with 3 of these recruits having died in the intervening period, leaving a total of 387 individuals alive. When considering only natural populations at Welbedacht, Skerpioenspoort and Middelberg, 597 individuals (73%) out of 821 had died, leaving only 264 trees alive.

For natural stands, there was no significant change in the average number of trees recorded in each photograph between early (8.65 ± 0.95, n = 51) and middle (6.86 ± 0.83, n = 28) (p = 0.787) periods. However, there was a significant decline (p < 0.001) in trees per photograph for the recent period (2.71 ± 0.34, n = 79). The early period had a significantly greater proportion of juveniles (27%) than the middle (5%) (Χ^2^ = 54.29, df = 1, *p* value < 0.001) and recent period (8%) (Χ^2^ = 36.68, df = 1, p value <0.001). However, no significant change was found in the proportion of juveniles between the middle and recent periods (Χ^2^ = 1.27, df = 1, p value = 0.259).

### Regional population dynamics

**Fig. 3:**
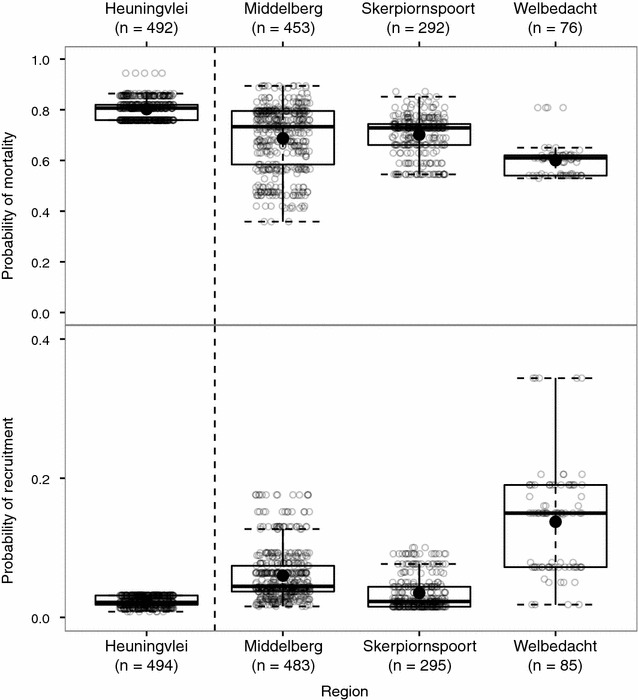
Modelled probability of *Widdringtonia cedarbergensis* mortality (Model 1) and recruitment (Model 2) at all sampled regions. Raw predicted points with *Tukey boxplots* indicating the median, lower and upper quartiles, and minimum and maximum values with outliers outside the* whiskers*. Means are shown as *filled black dots*

The plantation at Heuningvlei had the highest probability of mortality (mean ± SD: 0.90 ± 0.04) followed by the natural populations at Skerpioenspoort (0.70 ± 0.08) and Middelberg (0.69 ± 0.13) and then Welbedacht (0.60 ± 0.06) (Fig. 3). Welbedacht had the highest probability of recruitment (0.13 ± 0.08), followed in order by Middelberg (0.06 ± 0.04), Skerpioenspoort (0.04 ± 0.02) and lastly Heuningvlei (0.02 ± 0.01) (Fig. 3).

### Environmental trends

**Fig. 4:**
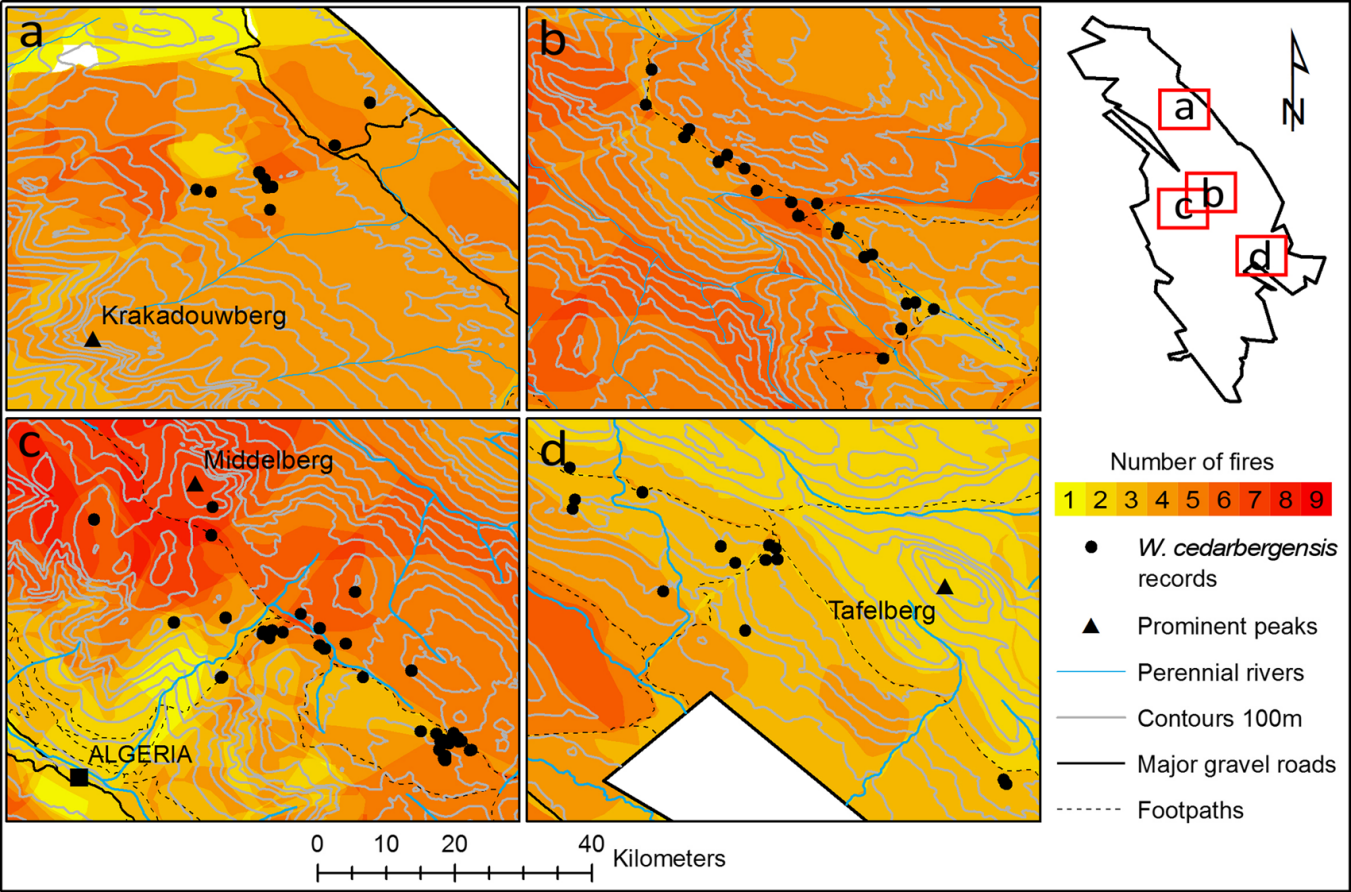
Fire frequency map for the four studied regions within the Cederberg Wildnerness Area. **a** Heuningvlei, **b** Skerpioenspoort, **c** Middelberg and **d** Welbedacht. The fire record is from 1944 to 2012

The range in fire frequency for all photo-sites was between three and eight fires over the recorded period (1944–2012). Middelberg experienced the highest median fire frequency of five fires, while Welbedacht experienced the lowest median of three fires (Fig. 4). Frequency histograms indicated mixed trends in the lengths of fire return intervals over time at the respective populations. Only Middelberg displayed a generally decreasing fire return interval (*i.e.* more frequent fires) through time (Additional file 4: Figure S2).

**Fig. 5:**
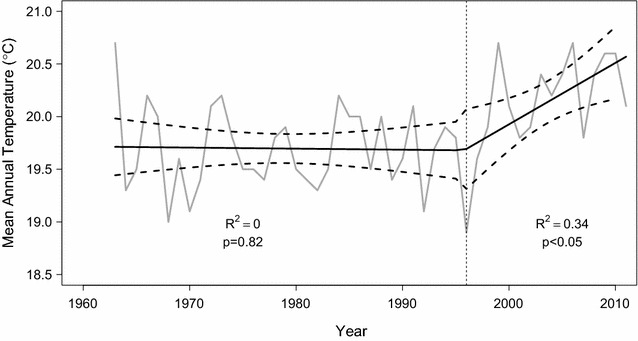
Mean annual temperature (°C) in Clanwilliam from 1963 to 2010. The* solid line* represents a piecewise regression with an estimated breakpoint in 1996. The* dashed lines* represent 95% confidence intervals. R^2^ values and significance levels are attached

Despite high inter-annual variability, precipitation increased significantly from 1908 to 2008 at Algeria (p < 0.001), but showed no significant trend at Clanwilliam only 25 km away (Additional file 5: Figure S3). The ten year mean rainfall amount was also significantly higher (636 ± 51 mm) at Algeria relative to Clanwilliam (177 ± 17 mm) (df = 198, F = 564, p < 0.001). There was a significant increase (p < 0.05) in temperature of approximately 0.6 °C at Clanwilliam over the recording period with segmented regression indicating that most of this increase occurred after 1996 (Fig. 5).

### Correlates of *W. cedarbergensis* tree mortality

**Fig. 6:**
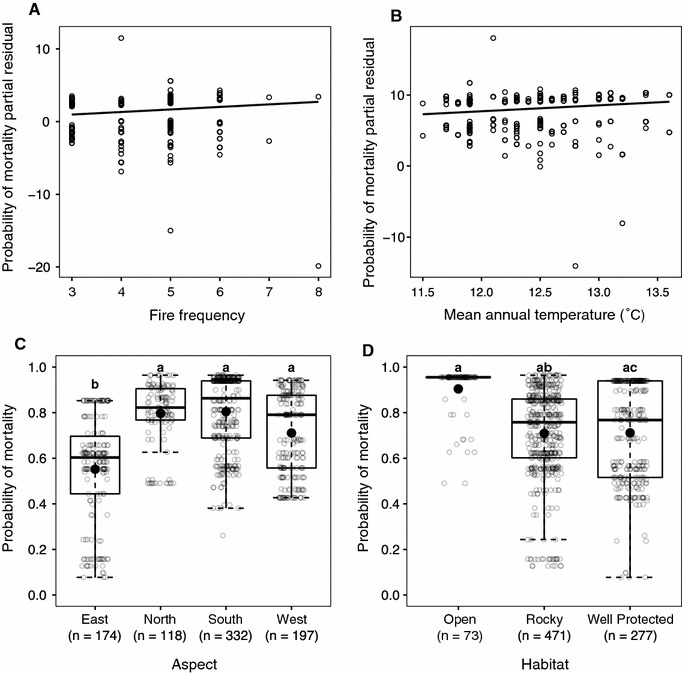
**A**–**D** Partial *residual plots* (**A**, **B**) and *predicted points* (**C**, **D**) showing the probability of *Widdringtonia cedarbergensis* mortality (Model 3) only natural populations in relation to environmental and climatic variables. *Regression lines* indicate the partial fit for **A** fire frequency and **B** mean annual temperature; and raw predicted with *Tukey boxplots* for **C** aspect and **D** *habitat* indicate the median, lower and upper quartiles, and minimum and maximum values with outliers outside the whiskers. Means are shown as filled *black dots*.* Different letters* indicate where the modelled probability of mortality was significantly different between aspects or habitat (p < 0.05)

Fire frequency, aspect, degree of rockiness and mean annual temperature all had a significant effect on *W. cedarbergensis* mortality (p < 0.10) (Table 1: Model 3). Trees in ‘open’ habitats did not have an increased probability of mortality (Table 1), although a significant difference in the abundance of *W. cedarbergensis* trees in rocky (n = 471), compared to well protected (n = 277) and open (n = 73) sites was observed (df = 2, Χ^2^ = 289.47, p < 0.001). In most cases the model captured the expected direction of relationships between the response and explanatory variables. For example, the probability of mortality increased with an increase in fire frequency (p < 0.05), while an increase in mean annual temperature was almost significant at α = 0.05 (p = 0.07) (Fig. 6). The predicted probability of mortality was greatest on northern, southern and western facing aspects, followed by eastern aspects (Fig. 6). Both elevation (positive) and annual precipitation (negative) had strong co-correlation with mean annual temperature (R^2^ = 0.59, p < 0.001 and R^2^ = 0.42, p < 0.001, respectively).

**Table 1:** Fitted coefficients, the standard errors, *P* values of the generalized linear mixed-model for probability of *W. cedarbergensis* mortality across all regions (model 1) and in natural stands (model 3), probability of recruitment across all regions (model 2) and the Wald test of fixed effects

Predictors	Model 1		Model 2		Model 3	
	Estimate	S.E.	Estimate	S.E.	Estimate	S.E.
(a)						
Intercept	–9.14*	2.85	9.23	5.72	–9.96*	4.40
Habitat-open	0.39	0.45	1.19	0.76	0.41	0.61
Habitat-rocky	0.26	0.23	0.38	0.50	0.55*	0.27
Aspect-north	1.45***	0.41	0.00	0.82	1.99***	0.45
Aspect-south	0.49	0.30	0.15	0.69	1.24***	0.34
Aspect-west	0.89**	0.33	0.78	0.63	1.43***	0.41
Fire frequency	0.09	0.12	–0.18	0.23	0.30*	0.14
MAT	0.70**	0.23	–0.96*	0.47	0.64	0.36
Random effects (R.E.)	0.00	0.97	0.00	0.98	1.02	0.90

Predictors	Χ^2^	*p* value	Χ^2^	*p* value	Χ^2^	*p* value
(b)						
Habitat	18.6	0.000	2.5	0.290	9.0	0.029
Aspect	18.8	0.000	8.7	0.068	26.9	0.000
Fire frequency	0.5	0.468	0.6	0.440	4.6	0.033
MAT	9.6	0.002	4.2	0.041	3.2	0.073

R.E.: n_region_ = 4; n_site_ = 86, R.E.: n_region_ = 4; n_site_ = 86, R.E.: n_region_ = 3, n_site_ = 80, n_tree_ = 1313, n_tree_ = 1357, n_tree_ = 821

Habitat well protected and aspect–east set as intercept, *MAT* Mean annual temperature

*p* values: 1 < 0.10, * < 0.05, ** < 0.01, *** < 0.001

## Discussion

The Cederberg was declared a Wilderness Area in 1973 giving it the highest conservation status in South Africa [31]. Grounded in the perception that *W. cedarbergensis* is in decline [6, 7], one of the objectives for the reserve has been the conservation of this iconic tree. Palaeoecological evidence does indeed suggest that *W. cedarbergensis* trees were once more numerous, with a steady decline in fossil pollen deposits since the last glacial maximum [10]. The suggestion is that the decline may be attributed to long-term warming and drying of the West Coast climate in the late Quaternary that may have contributed to elevated fire frequencies [10]. It has also been proposed that prior to heavy 18th and 19th century logging [5, 6] the species may have occurred in denser communities. Harvesting and more frequent anthropogenic fires led to thinning and reductions in stand size, thereby establishing a positive feedback which promoted further penetration by fires, killing both seedlings and large seed-bearing adults [9].

Without access to accurate demographic records it is very difficult to reconstruct population dynamics before the 20th Century. Our more recent results suggest, however, that population densities were much higher in the early part of last century than they are today and that the *W. cedarbergensis* decline has continued regardless of any management interventions [13, 32]. In addition, our results show that the greatest declines in trees took place between the periods 1960–1987 and 2007–2013, indicating that *W. cedarbergensis* mortality has increased in the late 20th and early 21st century. Current recruitment rates are also not keeping pace with mortality at any of our study sites. Importantly, the spatial consistency of high mortality and low recruitment across geographically isolated populations suggests that the factors driving the decline act over the entire range of the species.

Our model identified increased fire frequency and possibly higher temperatures as the primary drivers for *W. cedarbergensis* mortality. There are more trees still alive at higher elevations with concomitant lower temperatures and greater rainfall. It has been demonstrated that a small increase in temperature can increase vapour pressure deficit (VPD) which increases tree water use resulting in an increase in mortality during severe drought due to xylem cavitation [33]. This increased mortality with increased temperature is also demonstrated by our model showing a significant increase in mortality on warmer north facing slopes.

Although our modelled results suggest *W. cedarbergensis* mortality is not associated with more open habitats, we found that *W. cedarbergensis* trees were positively associated with ‘rockier’ sites. This suggests a variable not considered in the analysis but weakly correlated to the degree of rockiness such as, for example, available ground water, may be important in *W. cedarbergensis* survival. Previous research has demonstrated that in rocky sites the trees source water from deep pockets between the rocks [11]. This implies a possible affinity for available water. In addition, directed secondary dispersal [34] and shadier, cooler microhabitats found in rockier environments which are beneficial in seedling establishment [3] are also likely factors that have influenced recruitment and the presence of the trees in such sites.

Temperature-induced mortality has been recognised as a global issue for many tree species [1, 2, 35, 36]. The association of *W. cedarbergensis* trees with sites of lower temperatures at higher elevations and historical trends of increasing temperatures in the Cederberg, should be recognised as possible contributors to mortality. Not even a significant increase in rainfall over the course of the 20th century at the more proximal climate station at Algeria appears to have halted or reversed the negative demographic trend. Climate models for the study area [18] and globally [37] show that temperatures will continue to increase through this century, suggesting that in future favourable habitat for the trees is only likely at higher elevations [38]. Current populations of *W. cedarbergensis* are already at or near the highest locally attainable elevations. In addition, isolated peaks and ridges on which the trees typically occur are often separated from the nearest adjacent higher ground by lower-lying valleys dominated by fire-prone vegetation.

Given the slow rate of *W. cedarbergensis* growth to reproductive maturity, the median fire frequencies at the sampled sites over the 68 year record for the Cederberg suggests that fire return intervals will likely be too short in most regions for the trees to naturally replace themselves [3]. Indeed, in some regions fire return intervals are *decreasing* despite a management policy of a 15–20 year return interval. Our study did not, however, determine the relative impact of less frequent, large, high intensity fires (e.g. in 1959, 1985 and more recently in January 2013), on *W. cedarbergensis* demography and this remains an avenue for further research . Furthermore, future studies may benefit from the use of climatic and environmental variables with a more detailed temporal and spatial resolution, particularly in view of the myriad micro-habitats and refugia that are available in terrain such as exists in the CWA.

Despite the Cederberg being declared a Wilderness Area in 1973 and one of the management foci being the conservation of the threatened *W. cedarbergensis*, our results show that there has been a significant decline in the number of trees since that time. We suggest that this continued decline is probably due to both natural and anthropogenically mediated increases in fire frequency and temperature. If modelled climate change predictions for increased warming and drying of the Cederberg Mountains [18] are realised, the future survival of the *W. cedarbergensis* is at serious risk.

## Conclusions

The outlook for *W. cedarbergensis* remains a concern under the current climate and fire regime. Mortality rates are too high and recruitment rates too low to sustain viable populations in the long-term. The main drivers of mortality appear to be higher temperatures and shorter fire return intervals, while high elevation, rocky, east-facing environments represent refugia that provide fire protection, more reliable groundwater [11] and cooler microhabitats conducive to seedling establishment. Projected drying and temperature increases in the 21st century [18] suggest that the species will be under increasing pressure, both from the impacts on water balance and hydraulic failure under greater water stress, and from elevated fire frequencies.

However, there are interventions that could help sustain current populations or the establishment of new ones. Already established annual seedling out-planting programmes could be improved to increase *W. cedarbergensis* seedling survival. This could be done by incorporating beneficial criteria for *W. cedarbergensis* survival, as outlined in this study, when selecting sites for out-planting [32]. Of the populations which remain, a subset could be selected (based on current demographic profiles, elevation and temperature, degree of rockiness, etc.) for special protection from fire. Lastly, assisted migration [39] to more southern high-lying protected areas should be a serious consideration given the time it would take for viable populations to establish in such areas.

## Additional files

**Additional file 1: Table S1.** Details of photographers for all 87 repeat photograph photo-pairs taken in the Cederberg Wilderness Area between 1931 and 2013.

**Additional file 2: Table S2.** Source, spatial and temporal resolution, and unit of measurement of environmental variables.

**Additional file 3: Figure S1.** Moran’s *I* plots testing for spatial autocorrelation in model residuals. Spatial autocorrelation in generalized linear mixed-effects model residuals a) without a spatial autocovariate term and b) with a spatial autocovariate term. Significant spatial autocorrelation (*p* < 0.05) at each distance lag is shown by black dots.

**Additional file 4: Figure S2.** Histograms of fire histories for each region. a) Heuningvlei, b) Skerpioenspoort, c) Middelberg and d) Welbedacht. The fire record is from 1944 to 2012.

**Additional file 5: Figure S3.** Annual precipitation (mm) in Algeria (1908 to 2008) and Clanwilliam (1870 to 2010). The solid lines represent regression lines. The dashed lines represent 95% confidence intervals. R2 values and significance levels are attached.

## Abbreviations

CWA: cederberg wilderness area
GLMM: generalized linear mixed-effects model
SD: standard deviation
RE: random effects
MAT: mean annual temperature
