# Supplementary figures and images for: Collapse of an iconic conifer: long-term changes in the demography of Widdringtonia cedarbergensis using repeat photography
Source: BMC Ecol. 2016 Nov 30;16:53. doi: 10.1186/s12898-016-0108-6 (PMC5129610; doi:10.1186/s12898-016-0108-6)

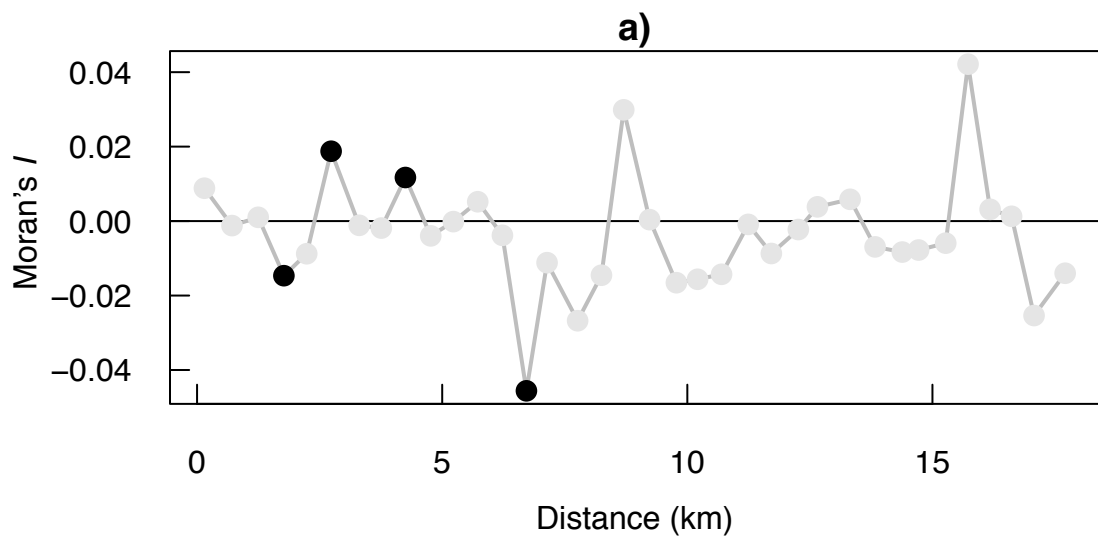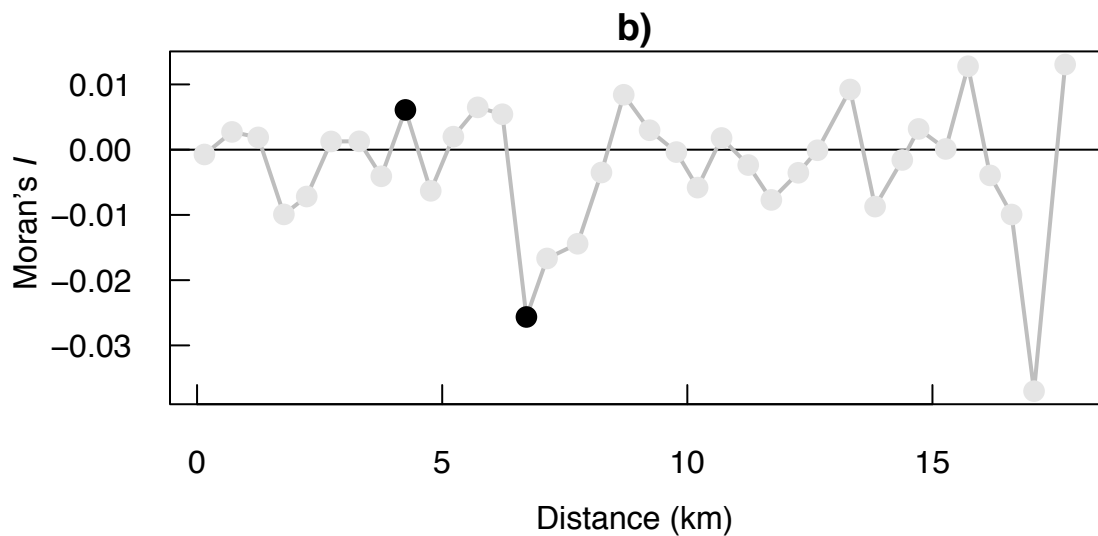

Supplement: Supplementary file 3 — Additional file 3: Figure S1. Moran’s I plots testing for spatial autocorrelation in model residuals. Spatial autocorrelation in generalized linear mixed-effects model residuals a) without a spatial autocovariate term and b) with a spatial autocovariate term. Significant spatial autocorrelation (p < 0.05) at each distance lag is shown by black dots. [file 12898_2016_108_MOESM3_ESM.pdf]

**a)**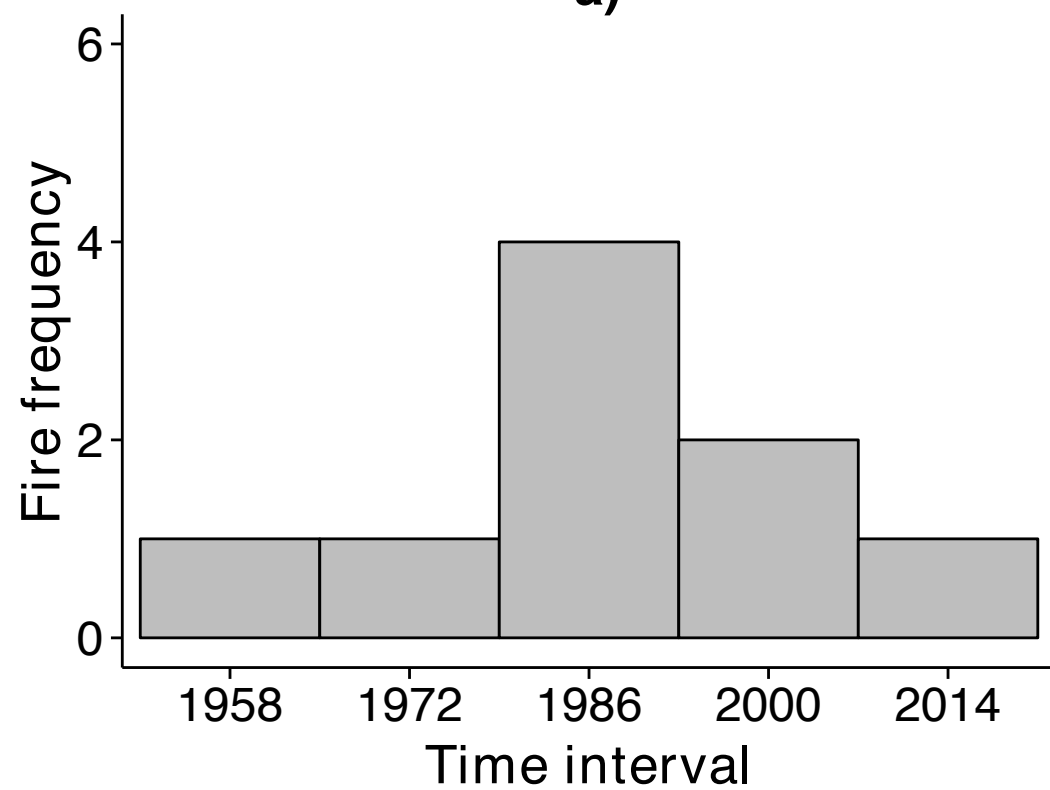**b)**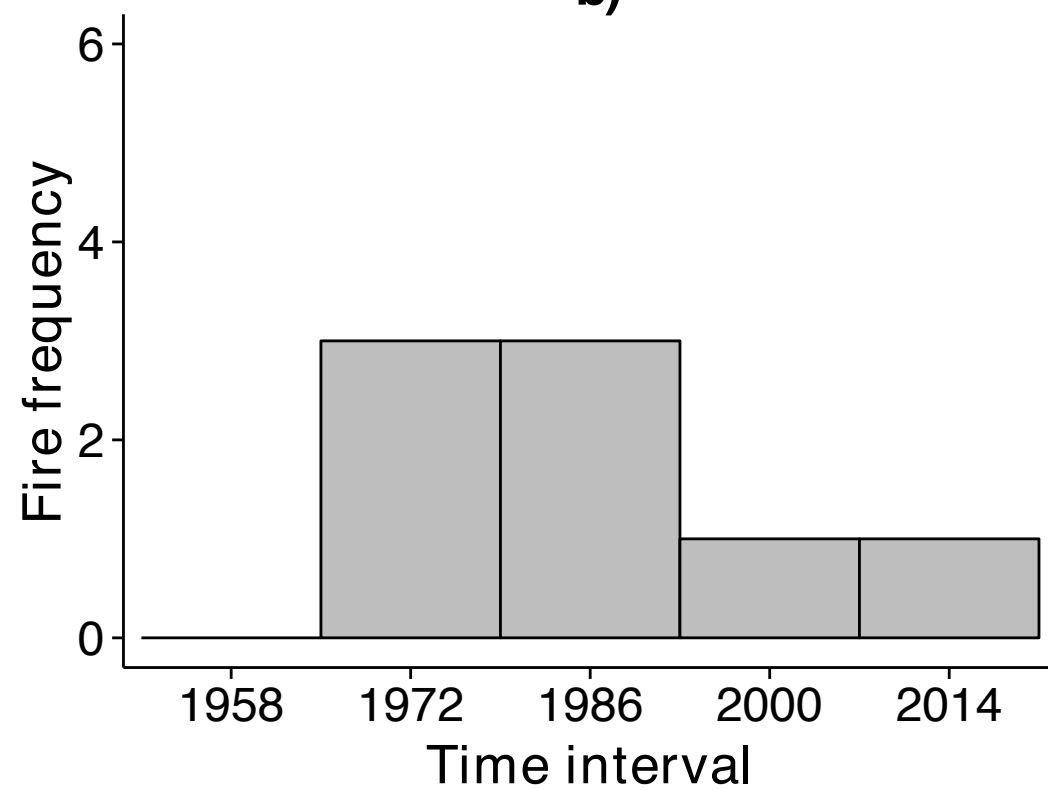**c)**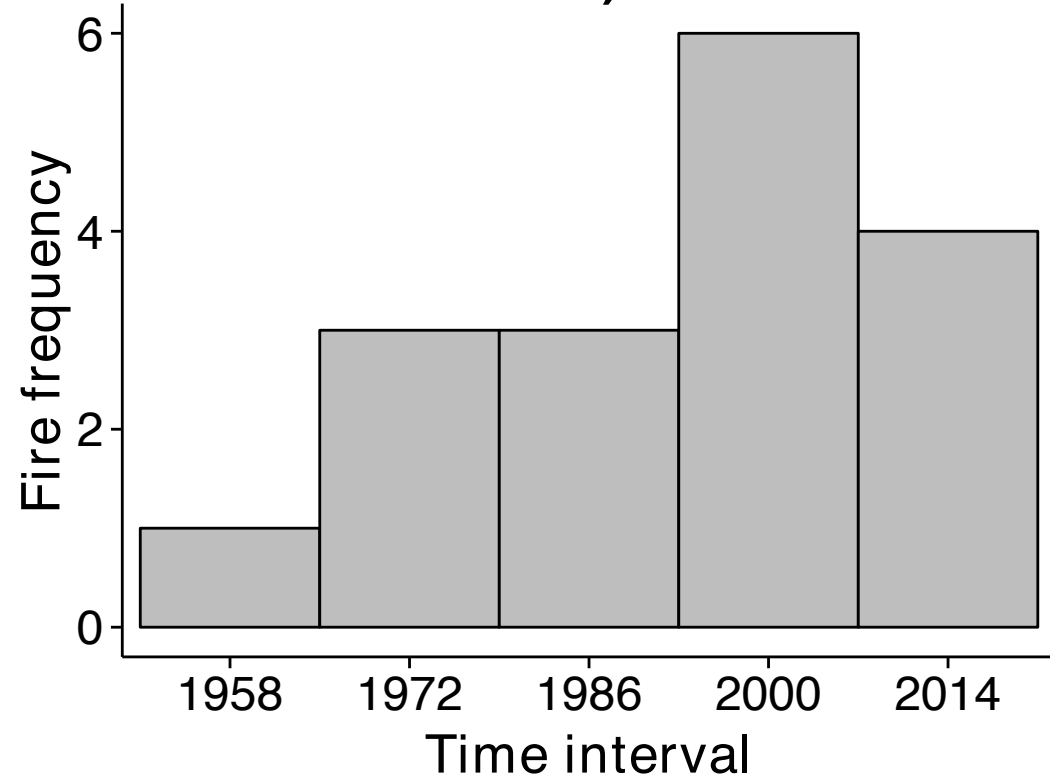**d)**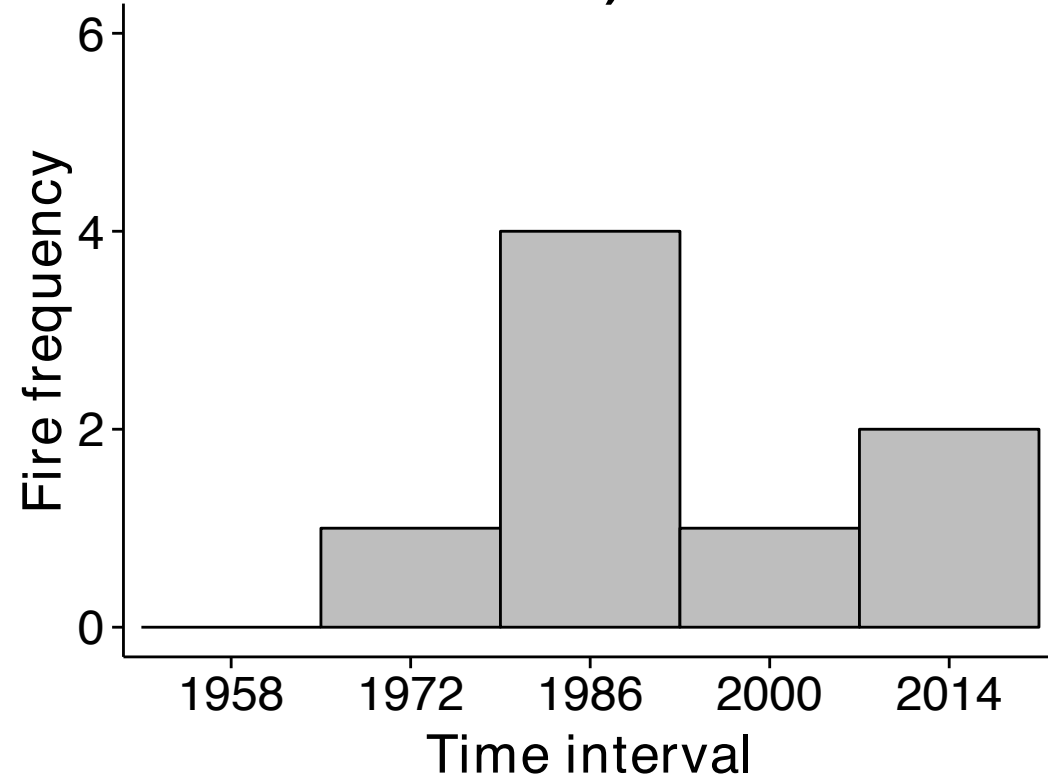

Supplement: Supplementary file 4 — Additional file 4: Figure S2. Histograms of fire histories for each region. a) Heuningvlei, b) Skerpioenspoort, c) Middelberg and d) Welbedacht. The fire record is from 1944 to 2012. [file 12898_2016_108_MOESM4_ESM.pdf]

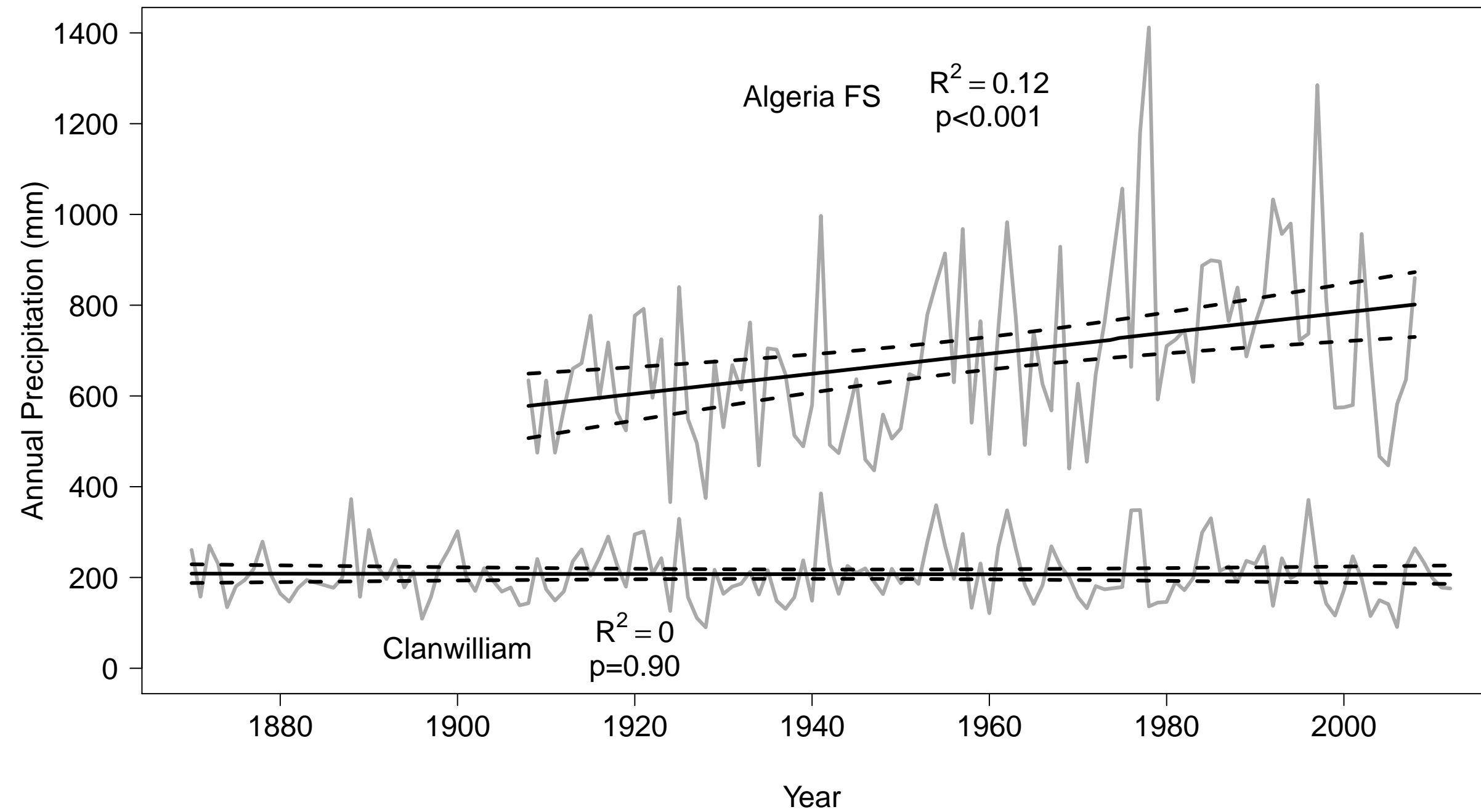

Supplement: Supplementary file 5 — Additional file 5: Figure S3. Annual precipitation (mm) in Algeria (1908 to 2008) and Clanwilliam (1870 to 2010). The solid lines represent regression lines. The dashed lines represent 95% confidence intervals. R2 values and significance levels are attached. [file 12898_2016_108_MOESM5_ESM.pdf]
